# Souveränität und Verantwortung im Mehrebenensystem: Subsidiarität als Leitmotiv?

**DOI:** 10.1007/s10273-021-2940-3

**Published:** 2021-06-22

**Authors:** Michael Hüther, Markus Vogel

**Affiliations:** 1 Institut der deutschen Wirtschaft e.V., Konrad-Adenauer-Ufer 21, 50668 Köln, Deutschland; 2 E1 Grundsatzfragen und Koordinierung der Europapolitik, Hessische Staatskanzlei, Georg-August-Zinn-Str. 1, 65183 Wiesbaden, Deutschland

**Keywords:** H77, K40, N44

## Abstract

Der Subsidiaritätsgedanke sollte von den politischen Entscheidungstragenden sowie von im europäischen Mehrebenensystem mitwirkenden Akteur:innen beachtet werden. Dazu gehört die Beachtung bei der legislativen Planung, dass die Einhaltung des Subsidiaritätsprinzips bedingungslose Voraussetzung ist. Zudem sollten alle an der europapolitischen Willensbildung Beteiligten für die Wahrung des Subsidiaritätsprinzips eintreten, wenn hieran Zweifel bestehen. Die Debatte um Anspruch und Wirklichkeit des europäischen Subsidiaritätsgedankens begleitet uns seit vielen Jahren, ist aber aktueller denn je.

## Abstract (en)

In the German political debate, the principle of subsidiarity plays an important role in the acceptance of the European multilevel system. In such a system, there is a collision of historically diverging, systematically inconsistent and qualitatively different needs. This combination demands an “active subsidiarity” and a “subsidiarity routine” in order to dynamically shape the heterogeneous EU. This way, we may find a balance between the needs of the citizens (acceptancy), the EU institutions (efficiency) as well as of the different levels of decision making and acting (reason for being). Above all, subsidiarity must start at home, it can be strengthened in the EU if it does not erode in the member states. This challenge has been regularly neglected til now.

Subsidiarität – an diesem Begriff, so technisch und sperrig einerseits, so schillernd und unbestimmt andererseits, kommt man in der europapolitischen Debatte nicht vorbei. Als in den europäischen Verträgen verankertes allgemeinpolitisches und rechtliches Leitprinzip sollte der Subsidiaritätsgedanke sowohl von den politischen Entscheidungstragenden als auch von allen im europäischen Mehrebenensystem mitwirkenden Akteur:innen regelmäßig beachtet werden. Die Debatte um Anspruch und Wirklichkeit bezogen auf den europäischen Subsidiaritätsgedanken begleitet uns seit mindestens dreißig Jahren. Sie ist aber aktueller denn je und wird in der Zukunftsdebatte eine große Rolle spielen (Hüther und Vogel, 2020).

Im Krisenmanagement zur Bewältigung der COVID-19-Pandemie zeigt sich, dass subsidiäre Ansätze die schnelleren Erfolge liefern (Coatleven et al., 2020). Zumindest werden Plädoyers für subsidiäre Ansätze zur Krisenbewältigung laut und finden Gehör. Etwa zeitgleich – in der medialen Aufmerksamkeit überlagert durch die Pandemiebekämpfung – verzichten zahlreiche subsidiaritätsrügeberechtigte nationale Parlamente in einem Fall, in dem die Einhaltung der Subsidiarität objektiv auf große Zweifel trifft, wohl aus politischen Erwägungen darauf, ihr Rügerecht zu nutzen.[1] Die Rede ist vom EU-Richtlinienentwurf für angemessene Mindestlöhne (Europäische Kommission, 2020a), die zu einem Kernvorhaben der Kommissionspräsidentin von der Leyen zählt und im Fokus ihrer Wahl im Juli 2019 stand.

Unabhängig von der Befürwortung bestimmter politischer Maßnahmen muss es bei der rechtlichen und organisatorischen Verfasstheit der Europäischen Union (EU) eine Rolle spielen, wer was und wie regeln darf. Wenn die Wächter der Subsidiarität ihrer Aufgabe nicht nachkommen (wollen), verpufft jeder Ruf nach aktiver Subsidiarität. Es wächst das Risiko, dass die Bürger:innen ohne Orientierung zurückbleiben. Das ist nicht ohne Gefahr, vor allem in Zeiten, die als unübersichtlicher denn je wahrgenommen werden.

Die EU hat allen Befürchtungen zum Trotz immer noch hohe Zustimmungswerte in der Bevölkerung. Unvermindert hoch ist aber der Druck, sich neben der Überwindung der Pandemie den drängenden Zukunftsthemen – wie Sicherheit, Migration, Digitalisierung, Innovation, Rechtsstaatlichkeit sowie Klimawandel – zuzuwenden. Besondere Erwartungen an weiterführende Impulse liegen auf der geplanten Zukunftskonferenz, die – über zwei Jahre – einen interinstitutionellen Dialog über die Zukunft der EU unter Einbeziehung der Bürger:innen vorsieht. Nachdem der Europatag 2020 coronabedingt abgesagt werden musste, ist auch unter

* Der Beitrag gibt ausschließlich die persönliche Meinung der Autoren wieder. Dr. Berthold Busch, IW, danken wir für wertvolle Hinweise.

1 Der Deutsche Bundestag hat mit Blick auf den Koalitionsvertrag der Regierungskoalition aus CDU/CSU und SPD von der Rüge abgesehen; im Bundesrat erhielt die Rüge aufgrund der Stimmen der CDU/CSU-geführten Länder eine Mehrheit, wurde dann aber auf Drängen grüner und sozialdemokratischer Koalitionspartner von den meisten Ländern im Bundesratsplenum nicht unterstützt.

**Prof. Dr. Michael Hüther** ist Direktor des IW in Köln und Honorarprofessor an der European Business School.

**MR Dr. Markus Vogel** ist Referatsleiter in der Europaabteilung der Hessischen Staatskanzlei.

deutscher EU-Ratspräsidentschaft trotz aller Versprechungen kein Start erfolgt. Das Thema Subsidiarität und Verantwortung ist jedenfalls aktuell, sodass – auch wenn sich die EU-Institutionen nicht mehr dazu geäußert haben – damit zu rechnen ist, dass das Thema von den Bürger:innen in der Debatte eingefordert wird.

## Subsidiarität in der Kompetenzordnung

Das Subsidiaritätsprinzip hat in der deutschen Diskussion eine besondere Bedeutung. Aus der katholischen Soziallehre und dem Liberalismus des 19. Jahrhunderts stammend hat es seine Wirkung vor allem als Orientierung für die Verantwortungsteilung zwischen Selbstverantwortung und subsidiärer kollektiver Unterstützung entfaltet. Eine (staatliche) Aufgabe soll so weit wie möglich von der unteren Ebene bzw. der kleineren Einheit wahrgenommen werden; zentrale Begriffe sind Selbsthilfe und Selbstorganisation, notwendiger staatlicher Schutz sowie familiäre und genossenschaftliche Solidarität. Über den sozialpolitischen Diskurs und die Debatte über die Verantwortung im föderalen System ist der Grundsatz der Subsidiarität schließlich in die ökonomische Diskussion gelangt, auch als Ordnungsidee für die soziale Marktwirtschaft. Theoretisch referenziert dieser Grundsatz auf die räumlichen Allokationswirkungen unterschiedlicher Güterkategorien, um Verzerrungen durch Überlagerungen, Interdependenzen, Haftungsunklarheiten zu vermeiden und einen angemessenen Konnex zu den Finanzierungsentscheidungen sicherzustellen.

Ebenso bezieht sich das Subsidiaritätsprinzip juristisch auf das Verhältnis der föderalen Ebenen und hat so seinen Weg in die europäischen Verträge gefunden, zunächst in der Einheitlichen Europäischen Akte 1987 für den Bereich der Umweltpolitik, dann mit dem Maastrichter Vertrag 1992 als generelle Norm für die EU. Mit dem Lissaboner Vertrag 2009 wurde den nationalen Parlamenten zugewiesen, über die Einhaltung des Subsidiaritätsprinzips zu wachen; ihnen wurde somit eine Präventivkontrolle mittels Subsidiaritätsrüge sowie eine nachträgliche Klarstellung durch den Europäischen Gerichtshof mittels Subsidiaritätsklage eröffnet (Parlament der Republik Österreich, 2008).

Vom juristischen Standpunkt fungiert das Subsidiaritätsprinzip als Regulativ zur Kompetenzordnung, und zwar als verbindliches Rechtsprinzip des EU-Rechts (Bickenbach, 2013, 523 ff). Danach sind die Zuständigkeiten der EU nach dem Prinzip der begrenzten Einzelermächtigung beschränkt. Die EU darf nur in den Bereichen legislativ tätig werden, die ihr durch die Verträge ausdrücklich zugewiesen sind (Verbandskompetenz) und nur durch die dort genannten Organe (Organkompetenz). Erst bei Vorliegen einer Ermächtigung zu legislativen Maßnahmen kommt das in Art. 5 Abs. 3 EUV verankerte Subsidiaritätsprinzip ins Spiel und ist dann zwingend heranzuziehen.

Das Subsidiaritätsprinzip verleiht der EU keine Kompetenzen, sondern schränkt im Wege einer normativen Vorgabe auf Grundlage der begrenzten Einzelermächtigung vorhandene Kompetenzen ein. Man spricht hier von der Kompetenzausübungsschranke, die den Gestaltungsspielraum der Mitgliedstaaten und ihrer Untergliederungen garantiert und in jedem Fall der geteilten Zuständigkeit heranzuziehen ist, von allen an der Rechtsetzung beteiligten Akteuren. Im Mehrebenensystem ist es Aufgabe aller Ebenen, auf die Einhaltung des Subsidiaritätsprinzips zu achten.

Das Subsidiaritätsprinzip in Artikel 5 Abs. 3 EUV unterscheidet in der Frage, wer für die Ausübung einer Kompetenz zuständig ist, nur zwischen den Mitgliedstaaten und der Gemeinschaftsebene. Dass aber „die Maßnahmen der Europäischen Gemeinschaft gemäß dem Subsidiaritätsprinzip nicht nur die Mitgliedstaaten betreffen, sondern auch deren Gebietskörperschaften, soweit diese nach nationalem Verfassungsrecht eigene gesetzgeberische Befugnisse besitzen“, hat die – vom Amsterdamer Gipfel zur Kenntnis genommene – „Erklärung Deutschlands, Österreichs und Belgiens zur Subsidiarität“ zum Ausdruck gebracht (Landtagspräsidentenkonferenz, 2017).

Die wichtigste Option ist hier die Erhebung einer Subsidiaritätsrüge mitsamt begründeter Stellungnahme durch die nationalen Parlamente. Sie haben damit ein formales Recht, im europäischen Gesetzgebungsprozess zu einem frühen Zeitpunkt zu rügen, wenn die EU eine Materie regeln will, die nach ihrer Auffassung ausreichend auf mitgliedstaatlicher Ebene geregelt werden kann. Zwar mangelt es nicht an Subsidiaritätsrügen,[2] doch wurde das notwendige Quorum erst in drei Fällen[3] überschritten. Die Handhabung der Subsidiaritätsrüge ist in der Praxis durch unterschiedliche Rechtsauffassungen und politische Präferenzen geprägt. Kompliziert wird die Situation dadurch, dass unter den nationalen Parlamenten keine einheitliche Auffassung darüber herrscht, wie weit die Subsidiaritätsrüge gehen kann: Ist diese streng juristisch auf die Rüge eines Verstoßes gegen das Subsidiaritätsprinzip beschränkt oder umfasst sie auch Verstöße gegen den Verhältnismäßigkeitsgrundsatz sowie gegen das Prinzip begrenzter Einzelermächtigung (weiter politischer Subsidiaritätsbegriff)[4]? Jedenfalls bleibt das Thema Subsidiarität – wie es die Rüge im Mindestlohnfall erkennen lässt – bedeutsam in der eu-

2 Bisher 442 Rügen (14 vom Bundesrat, 11 vom Deutschen Bundestag)
3 Bezüglich „Monti II“ COM(2013)130, „Europäische Staatsanwaltschaft“ COM (2013)534 und „Entsenderichtlinie“ COM(206)128.
4 Der Bundesrat nutzt für die Prüfung eine Handreichung (21. Januar 2013), die auch Spielraum für unterschiedlich weite Auslegungen vorsieht. Nach Ansicht der Mehrheit der Länder (vornehmlich B-Länder) ist eine tragfähige Kompetenzgrundlage als notwendige Vorfrage Bestandteil der Subsidiaritätsprüfung; einige A-Länder wollen das Fehlen jeglicher Kompetenz im Rahmen des politischen Dialogs und damit außerhalb des Frühwarnsystems verorten.

ropapolitischen Befassung der nationalen Parlamente, insbesondere beim Bundesrat (Deutscher Bundesrat, 2019).

In dem Mehrebenensystem EU kollidieren historisch divergierende, systematisch inkonsistente und qualitativ unterschiedliche Bedürfnisse, die auszugleichen sind. So besteht die Herausforderung, ein differenziertes und gleichzeitig demokratisches Mehrebenensystem zu gestalten. Während die Erwartungen an die völkerrechtliche Funktion des Subsidiaritätsprinzips mit präventivem Schutz der unteren Ebenen nicht erfüllt werden konnten (Hatje und Schwarze, 2019, 185) und die juristische Orientierung zwischen Verfassungsrecht sowie Völkerrecht zu leichtfertig einen Transfer mit Effektivitätshoffnung nahegelegt hat, eröffnet der ökonomische Diskurs mit der Theorie der Allmende eine interessante Orientierung. Die Theorie der Allmende (Hardin, 1968) bezieht sich auf ein Gut, bei dem der Konsum rivalisiert, aber ein Ausschluss von Nichtzahlenden unmöglich ist und es deshalb zur Übernutzung kommt (Ausbeutung freier Ressourcen). Der Staat ist deshalb gefordert, Nutzungsordnungen zu erlassen. Öffentlicher Raum, Öffentlichkeit und Sozialkapital können als Allmendegut gedeutet werden, indem man deren Bewirtschaftung durch mehrere staatliche Ebenen als Konkurrenz um die Wählerpräferenzen deutet, der es infolge der Komplexität an Transparenz fehlt. Das führt zu einem Koordinierungsversagen und dazu, dass die Wahlbürger:innen – quasi die Ressourcen der Allmende „öffentlicher Raum" – zunehmend indifferent werden; sie werden „übernutzt", und das Vertrauen in die staatlichen Institutionen droht zu schwinden. Dies entspricht im Grundsatz der Erosion von Allmendegütern.

Die grundsätzliche Dynamik des Mehrebenensystems darf zudem nicht übersehen werden. Insofern richtet sich die Orientierungssuche nicht auf einmalige, quasi definitive Systemlösungen, sondern auf Gestaltungsprinzipien, die den Ausgleich der verschiedenen historischen Pfade und systematischen Perspektiven unter sich wandelnden Bedingungen möglich machen. Folgende Orientierungen (Designprinzipien) lassen sich im Analogieschluss zum Allmendephänomen ableiten (Ostrom, 1990, 1999):

- Bei der Gestaltung eines Mehrebenensystems geht es um ein Regelwerk für die Klärung von Zuständigkeiten und Verantwortlichkeiten. Ein konsistentes Regelwerk beruht auf der Bottom-up-Perspektive, sichert institutionelle Kongruenz sowie fiskalische Äquivalenz, sodass sich Handlungskompetenz und -legitimation sowie Finanzierungsverantwortung entsprechen. Dadurch entsteht Transparenz über die Wirksamkeit unterschiedlicher Ebenen der Staatlichkeit für die Bürger:innen.

- Dazu gehören auch die Klärung gemeinschaftlicher Aufgaben, das Monitoring der realen Politik hinsichtlich der definierten Ordnung, der Definition automatischer Sanktionen bei institutionellem Fehlverhalten (Subsidiaritätsverstoß) sowie der Festlegung von Mechanismen zur Konfliktlösung. Denn realistischerweise ist angesichts lebenswirklicher Komplexität nicht davon auszugehen, dass bei aller Konsistenz des Regelwerks eine vollständige Überschneidungsfreiheit zu realisieren ist.

- Es müssen institutionelle Anpassungsverfahren bei sich verändernden politischen, sozialen und technischen Bedingungen des Mehrebenensystems etabliert werden, um der Dynamik des Umfeldes Rechnung tragen zu können. Besonders für Krisen transnationaler Qualität – wie in der Pandemie – sind gesonderte Entscheidungsstrukturen vorzusehen, die ein Auseinanderfallen der Ebenen und Widersprüche im jeweiligen Handeln vermeiden, zugleich die notwendige Kooperation sicherstellen (z. B. beim Grenzmanagement).

## Suche nach Orientierung für eine bessere Balance der Verantwortlichkeiten

Der Blick in die juristische und ökonomische Debatte zum Subsidiaritätsprinzip bleibt dennoch unbefriedigend. In diesem Mehrebenensystem kollidieren – wie angeführt – historisch divergierende, systematisch inkonsistente und qualitativ unterschiedliche Bedürfnisse, die in einen Ausgleich zu bringen sind. Für die EU besteht die Herausforderung, ein differenziertes und gleichzeitig demokratisches Mehrebenensystem zu gestalten und zu leben. Dabei wissen wir möglicherweise nicht eindeutig, wo die angemessene Aufgabenzuordnung in diesem gewachsenen Mehrebenensystem liegt. Denn die Unübersichtlichkeit im europäischen Mehrebenensystem macht es nicht nur Außenstehenden schwer, die Beiträge der jeweiligen Ebene zur Formulierung einer konkreten Politik überhaupt zu identifizieren. Tatsächlich sind bereits Misch- und Verbundlösungen – in den Mitgliedstaaten – realisiert, die Komplexitäten begründen, schwerfällig sind, Transparenz erschweren und Plausibilitäten überdecken. Es folgen Akzeptanzprobleme, die eine EU der Zukunft am wenigsten gebrauchen kann.

Zentral für die Frage, welche konkreten Gestaltungsprinzipien am besten den erhofften Ausgleich bringen können, ist der Blick auf die Bedürfnisse (vgl. Abbildung 1), und zwar

- die Bedürfnisse der Bürger:innen in ihren jeweiligen Lebenskontexten und Sachbezügen (Akzeptanz durch Mehrwert);
- die Bedürfnisse der verschiedenen einzelnen Gestaltungs- und Entscheidungsebenen (Daseinsberechtigung durch Funktionalität);
- die Bedürfnisse der EU (Wirksamkeit und Effizienz).

Um zu besseren Einordnungen zu gelangen, ist zugleich die Heterogenität in Europa in den Blick zu nehmen. Was in einem Mitgliedstaat als Belastung, Problem oder Hürde in der Politikgestaltung angesehen wird, mag in einem anderen Staat keine oder nur eine untergeordnete Rolle spielen. Die Feststellung von Heterogenität und Disparitäten legt ebenfalls nahe, dass es nicht die eine (einzig denkbare) systematische Lösung geben wird.

1. Der Subsidiaritätsgedanke unterscheidet sich sehr in der EU aufgrund *historischer und habitueller Differenzierungen* mit Blick auf die Verläufe von Staatenbildung und die Gesellschaftsmodelle. Das zeigt sich vor allem bei der Frage, ob primär die Legitimation zentraler Kompetenzen verfolgt wird oder es vordergründig darum geht, zentrale Kompetenzen einzuhegen oder abzuwehren. Ein wiederkehrendes Problem in der Praxis ist, dass es im Laufe der politischen Debatte um Subsidiarität häufig nur darum geht, ob eine Maßnahme auf EU-Ebene erlassen wird oder nicht. Dabei sollte geklärt werden, welcher Akteur auf welcher Ebene die Maßnahme erlässt.

2. Eine weitere Rolle spielen die *heterogenen Mehrebenenstrukturen*. In den Mitgliedstaaten herrschen große Unterschiede im staatsorganisationsrechtlichen Aufbau; insbesondere differiert stark, wie viele Ebenen am staatlichen Geschehen mitwirken, welche Ebenen das sind und wo deren Zuständigkeiten liegen. Dabei unterscheidet sich besonders, wie einzelne Ebenen an der Europapolitik beteiligt sind – bloße Umsetzung oder Mitwirkung als Region mit Gesetzgebungskompetenz – und nicht zuletzt, welche Rolle dieser Ebene in der landläufigen Wahrnehmung in Bezug auf die Europapolitik zugemessen wird. Spiegelbildlich zu diesen Einschätzungen variieren auch die Erwartungen der Bürger:innen an die jeweils bekannte Ebene.

3. Auch *Staatsaufbau und -verständnis* unterscheiden sich erheblich mit Bedeutung für die kulturelle, rechtliche und gesellschaftliche Identität. In allen 27 Mitgliedstaaten gibt es zwangsläufig unterschiedliche Erfahrungen mit Staatlichkeit, Kompetenzverteilung und Souveränität(en). Es gibt in der EU nicht nur die klassischen Föderal- und Einheitsstaaten als jeweilige Extreme, sondern auch Staaten, in denen der Regionalismus als Gestaltungsprinzip (in Italien eher schwach, in Spanien eher stark) gewählt wurde. Zudem unterscheiden sich unter den Einheitsstaaten auch die zentralistischen Einheitsstaaten (z.B. Finnland) von den dezentralen Einheitsstaaten (wie Frankreich, Dänemark oder Niederlande) (Sebaldt, 2009). Und nicht zuletzt gibt es in diesen Einheitsstaaten autonome Regionen, für die wiederum Abweichungen vom Grundprinzip gelten.[5] Innerhalb der EU waren 73 Regionen aus acht Staaten im Netzwerk REGLEG (Konferenz der Regionen Europas mit Gesetzgebungsbefugnis) aktiv;[6] zudem engagieren sich Regionalparlamente aus neun Staaten in der CALRE (Konferenz der Europäischen Regionalen Gesetzgebenden Parlamente).[7]

Abbildung 1
**Subsidiarität – Bedürfnisse und Plausibilitäten**

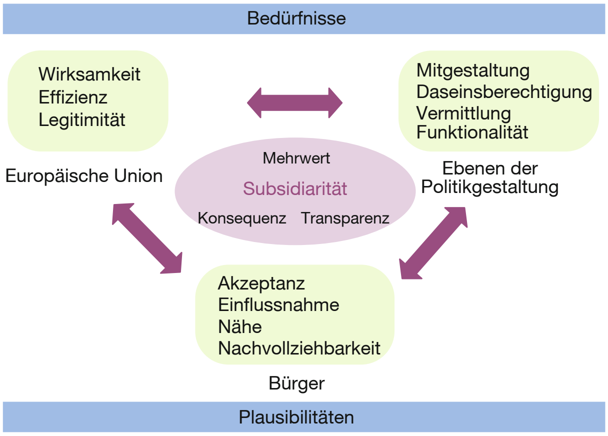

Quelle: eigene Darstellung.

4. Der Föderalismus gilt als bürgernah und flexibel, hat aber auch mit *limitierter Souveränität* zu tun. Mit überschaubaren Lebens- und Funktionsbereichen schafft der föderale Staat eine größere Bürgernähe und kann dazu beitragen, politische und administrative Entscheidungen auf ein menschliches Maß zurückzuführen. In föderalen Strukturen werden öffentliche Leistungen nach dem lokalen und regionalen Bedarf gewährt – ohne das Risiko zentralstaatlicher Fehleinschätzungen. Es gibt verschiedene Ebenen der Zuständigkeitsbereiche – auch in der Gesetzgebung –, die einander ergänzen. Aber Föderalismus ist kein Selbstläufer: Er muss für die Bürger:innen einen erkennbaren Mehrwert bringen, und er setzt die Bereitschaft zur erweiterten Verantwortungsübernahme voraus. Gerade durch Digitalisierung und E-Government sind die Grenzen föderaler Strukturen deutlich, weil nun die Prozesse unabhängig von institutionellen Zuständigkeiten im Mittelpunkt stehen. Ebenso hat die Pandemie die begrenzte, vor allem zeitraubende Umsetzungskapazität föderaler Systeme verdeutlicht.

5 Åland-Inseln, Azoren und Madeira.
6 Neben 16 deutschen und 9 österreichischen Bundesländern, 5 belgischen Regionen und Gemeinschaften auch 17 spanische Comunidades Autónomas, 20 italienische Regionen, die Countries Nordirland, Schottland und Wales des Vereinigten Königreichs sowie die Regionen Åland (Finnland), Azoren und Madeira (Portugal).
7 https://www.calrenet.eu/.

5. Nicht aus dem Blick geraten sollten *regionale Differenzierungsbedarfe*, die vor allem in Mitgliedstaaten ohne oder mit nur schwach ausgeprägten Föderalstrukturen zutage treten. Zu nennen sind Autonomiebewegungen und Regionalkonflikte, die es vielfach in der EU gibt und die teilweise (wie in Katalonien und Schottland) zu Sezessionsbewegungen avanciert sind. Aus EU-Sicht handelt es sich zwar um interstaatliche Angelegenheiten, für die die EU nicht zuständig ist. Interessant ist aber, dass aus den Lagern der Unabhängigkeitsbefürwortenden stets das Bekenntnis zur EU-Freundlichkeit hochgehalten und der Ruf nach einer „europäischen Lösung“ laut wird.

6. Nicht zuletzt sind die *territorialen Bezüge* von Interesse. Aufgrund gradueller Auflösungen von Staatsgrenzen erhalten räumliche Maßstabsebenen eine zunehmend höhere Relevanz (Löcse, 2019). Durch eine fortschreitende europäische Integration verliert der Nationalstaat in einigen Bereichen für die Bürger:innen an Bedeutung. Durch die grenzüberschreitende Zusammenarbeit sind transnationale Räume gewachsen, in denen Regelungsbedarfe nicht an der Grenze haltmachen, sondern darüber hinauswirken. Dort entstanden Europaregionen (Euregios), vornehmlich als überstaatliche Kommunalverbände organisiert. Häufig grenzt eine wirtschaftlich schwache Randregion an ein Wirtschaftszentrum jenseits der Staatsgrenze. Die Bedarfe der dort lebenden Menschen definieren sich vor Ort, und mit ihnen geht der Wunsch nach Überwindung administrativer und bürokratischer Hürden einher.

Der Blick auf diese Differenzierungsaspekte im Mehrebenensystem der EU bestätigt die These der vielgestaltigen Differenzierung. Wenn deshalb die Chance gering ist, weder ein einheitliches Verständnis der Subsidiarität noch eine systematisch gestaltete Ordnung der Subsidiarität für Europa zu finden, dann bleibt für die Gestaltung zunächst nur die Möglichkeit der *Perspektivenverankerung*, nämlich Bottom-up. Ein europäisches Mehrebenensystem sollte grundsätzlich von unten nach oben gedacht werden. Die konkrete Ausgestaltung der Zuständigkeit zwischen den Ebenen kann dann stärker den nationalen Traditionen folgen. Dazu erscheint der Vorschlag der Task Force vom 10. Juli 2018 passend, der das Konzept der aktiven Subsidiarität in den Mittelpunkt rückt (Task Force on Subsidiarity, Proportionality and „Doing Less More Efficiently“, 2018). Damit lassen sich die genannten Gestaltungsprinzipien und der Gesichtspunkt der Dynamik verbinden.

Um das Koordinierungsversagen in der „Allmende der europäischen Wahlbürger“ durch Übernutzung zu vermeiden, werden die institutionellen Ebenen der Staatlichkeit einem Koordinierungssystem unterworfen, das auf dem Gedanken beruht, dass Subsidiarität nicht nur gewährt wird, sondern permanent verlangt und praktiziert werden muss, quasi als *Subsidiaritätsroutine*. Das setzt einerseits voraus, dass die Aufgaben der EU klar bestimmt sind, um den subsidiaritätsfreien Bereich eindeutig zu definieren. Andererseits ist das verwaltungsmäßige Ineinandergreifen der Ebenen in jenen Bereichen, die einen europäischen Mehrwert vermuten lassen, gemäß der Subsidiaritätsroutine zu gestalten, zu beobachten und gegebenenfalls zu überprüfen und anzupassen.

Aktive Subsidiarität und Subsidiaritätsroutine führen dazu, dass das Miteinander im Mehrebenensystem in einen dynamischen Prozess eingebettet wird. Zugleich eröffnet diese Routine alle Differenzierungsmöglichkeiten, wie sie in Ansätzen vorhanden sind und kurz skizziert wurden: Umgang mit heterogenem Mehrebenensystem, mit differenzierten nationalen staatlichen Ordnungen oder der Eröffnung unterschiedlicher territorialer Bezüge. Die Routine folgt den Schritten: (1) Neue Handlungsoptionen, (2) umfassende Wirkungsanalyse, (3) Subsidiaritäts- und Verhältnismäßigkeitsprüfung, (4) Einbindung nationaler Parlamente, (5) koordinierter legislativer Prozess, (6) Umsetzung, (7) Evaluation.

## Plädoyer für Subsidiarität in der Zukunftsgestaltung

In der aktuellen Debatte um die Zukunft der EU müssen diese Perspektiven auf Subsidiarität sowie auf Strukturen und Verantwortlichkeiten im europäischen Mehrebenensystem in den Fokus rücken. Es sollte nicht nur um die Auswahl von politischen Schwerpunkten anhand von „europäischen Zukunftsthemen“ gehen, sondern vor allem um Überlegungen zu Aufgabenverortungen im Mehrebenensystem. Hierbei wird eine große Rolle spielen, welcher Spieler bzw. welche Institution was vorschlägt. Speziell sollten die politischen Akteure die ihnen zugedachte Rolle und Stellung im Mehrebenensystem kennen.

Aus der ökonomischen Theorie der Bürokratie lassen sich grundlegende Zweifel ableiten, ob die Europäische Kommission (EK) überhaupt die geeignete Instanz ist, über die Subsidiarität einer Maßnahme zu befinden. „Bürokraten wird – wie in der ökonomischen Theorie der Politik üblich – Maximierung ihres Eigennutzens unterstellt; an der Erreichung der offiziellen Ziele der Organisation sind sie nicht direkt interessiert“ (Frey, 1980, 663). Bürokrat:innen orientieren sich somit an Zielen wie „Macht, Prestige, Sicherheit und Einkommen“. Nach Niskanen wollen Bürokraten ihr Budget maximieren; Williamson zufolge nutzen Bürokraten einen diskretionären Spielraum aus, da sie nur unvollständig von Regierung und Wählern kontrolliert werden können (Frey, 1980, 664). So dürfte die EK eher an „mehr“ als an „weniger Integration“ interessiert sein und insofern auch dem Subsidiaritätsprinzip weniger Bedeutung zuzusprechen geneigt sein als der Europäische Rat und natürlich der Ausschuss der Regionen.

Mit der von der EK interinstitutionell gemeinsam mit Rat und Europäischem Parlament geplanten Konferenz zur Zukunft Europas, welche die Bürger:innen einbezieht, besteht die Chance, die hier angestellten Überlegungen in die Debatten einfließen zu lassen. Aus den vorliegenden Papieren von EK (Europäische Kommission, 2020b) und Parlament (Europäisches Parlament, 2020) lässt sich nicht herauslesen, inwieweit das Thema Subsidiarität eine Rolle spielen wird. Wenn man es aber ernst meint mit der Beteiligung der Bürger:innen, dann müssen vor dem Hintergrund ihrer Integrationsverantwortung auch die Regionen mit Gesetzgebungskompetenz sowie die nationalen Parlamente sowohl an der Organisation der Konferenz als auch an der Festlegung ihrer Ziele beteiligt werden und ähnlich wie bei der Einbeziehung in Arbeiten des Europäischen Konvents in den entsprechenden Planungsgremien repräsentiert sein.

Nur wenn föderale Strukturen und der Subsidiaritätsgrundsatz bei den Planungen zur Zukunft der EU berücksichtigt werden, kann die gewünschte Bürgernähe plausibel und das europäische Projekt bei den Menschen als positiv erfahrbar gemacht werden. Subsidiaritätsaspekte, Erfahrungen aus dem Föderalismus sowie Aspekte wie Territorialität und regionale Differenzierungsbedarfe sollten unbedingt mit im Zentrum von Zukunftsdebatten stehen. Denn die Frage der Verortung von Verantwortlichkeit ist kein Selbstzweck, sondern muss sich für die Bürger:innen erkennbar als vorteilhaft darstellen und plausibel sein. Andernfalls droht ein Akzeptanzverlust. Wer die Bürger:innen für die EU gewinnen will, muss vor allem das Subsidiaritätsprinzip ernstnehmen (Limbach, 2012).

Es liegt nahe, die Verantwortungsverteilung auf verschiedenen Ebenen beizubehalten sowie diese bei den weiteren Arbeiten an einer zukunftsfähigen EU stärker zu berücksichtigen, d.h. zu integralen Bestandteilen der Zukunftskonzepte zu machen. Denn die Vergrößerung von Gestaltungsräumen und die Stärkung der Eigenverantwortlichkeit auf den unteren Ebenen sind der beste Beitrag dafür, dass die EU von der Bevölkerung nicht als Fremdbestimmung empfunden wird. Schließlich gilt: Die Entscheidungsfindung auf verschiedenen Ebenen vermag die jeweilige Legitimität besser zu wahren und erhöht die Chance, dass Plausibilitäten sichtbar werden bzw. bleiben. Auch geben sie Raum für regionale Spezifitäten und bergen ein großes Potenzial, Regionalkonflikte einzudämmen und kleinzuhalten. Dazu gehört eine bedeutsame *Voraussetzung in den Mitgliedstaaten*: Es darf dort nicht zu einer internen Schwächung oder gar Aushöhlung der Subsidiarität kommen.

Wird die Subsidiarität im nationalen Rahmen geschwächt, dann erschwert das natürlich die Forderung an die europäische Ebene. Eine „aktive Subsidiarität“ der EU verlangt eine besonders aktive und „relevante Subsidiarität“ in den Mitgliedstaaten. Wer sich – wie die deutschen Länder – auf Mitwirkungsrechte beruft, muss auch die ihm national zur Verfügung stehenden Rechte ausüben oder auf deren Stärkung hinwirken. Das schließt kritische Fragen, wie die nach der angemessenen Mindestgröße einer Gebietskörperschaft, mit ein. Wenn aktive Subsidiarität ein größeres Verantwortungsbewusstsein und ein besseres Verständnis für die Politikgestaltung schaffen soll, dann muss damit die frühzeitige Beteiligung der unteren Ebenen an Folgenabschätzung und gegebenenfalls Gesetzgebungsverfahren einhergehen; deren Erfahrungen sind einzubeziehen und auch dort konsequent Subsidiaritätsprüfungen vorzunehmen. Subsidiarität hat dann das Potenzial, als Garant für ein „besseres Europa“ betrachtet zu werden und nicht als die Bremse für „weniger Europa“. Dazu gehört freilich ebenso, dass föderale Mitwirkungsansprüche nicht künstlich und willkürlich konstruiert werden, wo die Zuständigkeit – wie in der Handelspolitik – auf der europäischen Ebene liegt. Das kann als fehlgedeutete Subsidiarität das Prinzip nachhaltig schädigen.

Abbildung 2
**Subsidiaritätsstärkung**

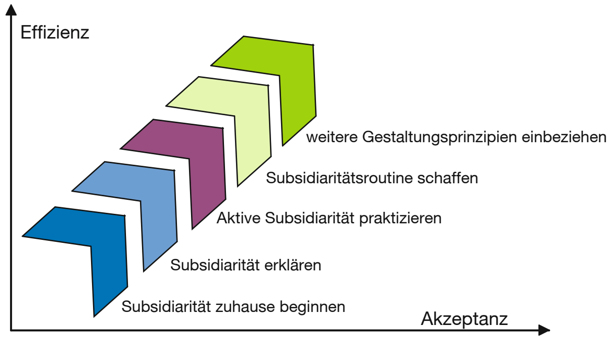

Quelle: eigene Darstellung.

Als nächste Schritte im Spannungsfeld von Effizienz und Akzeptanz sind somit zu gehen (vgl. Abbildung 2):

- *Subsidiarität neu erklären und einfordern*. Das in den Verträgen enthaltene Subsidiaritätsprinzip sollte – trotz aller Schwierigkeiten – künftig in einer Form gefasst sein, die dessen Justiziabilität als Kontrollmaßstab gewährleistet (Hatje und Schwartze, 2019, 164) und die Rückbindung an die nationalen Bedingungen herstellt, um eine Aushöhlung von unten zu verhindern. Die Rückwirkungen auf die nationalen Ordnungen sind dabei systematisch zu berücksichtigen. Wichtig ist die oft vernachlässigte Einsicht, dass die Subsidiarität in der EU nur aktiv gelebt und gestaltet werden kann, wenn sie im nationalen Kontext selbstbewusst entwickelt wird.

- *Ideen aus den Erfahrungen mit dem Föderalismus ableiten*. Welche Potenziale stecken in föderalen Strukturen? Welche Nachteile entstehen, wenn diese negiert werden? Die Potenziale, die für die EU in föderalen Strukturen liegen, sollten diskutiert werden, vor allem darf sich der Blick nicht verschließen auf die Nachteile, die entstehen, wenn föderale Bedürfnisse übersehen oder gar negiert werden. Ebenfalls muss laut gefragt werden (können), ob und gegebenenfalls welche Anpassungen der europäische Integrationsprozess mit Blick auf bestehende Mehrebenenstrukturen erfordert.

- *Über weitere konkrete Gestaltungsräume, Prinzipien und Prozeduren nachdenken*. Das kann sich auf neue Perspektiven für Euregios oder die stärker thematisch orientierte Kooperation von Spiegel-Regionen beziehen, nämlich solchen, die sich strukturell mit Blick auf relevante Faktoren (gesellschaftlich, kulturell, ökonomisch) ähneln. Das könnte auch eine Stärkung dieser Ebenen in den Nationalstaaten bewirken. Die Frage wäre zu klären, ob im europäischen Mehrebenensystem die Subsidiarität vor allem vertikal unter Berücksichtigung der bestehenden Verfassungsordnungen bedeutsam sein kann oder nicht auch stärker horizontal unter Berücksichtigung vergleichbarer Interessenlagen in einzelnen Mitgliedstaaten. Jedenfalls bedarf es einer Subsidiaritätsroutine, um dieses Grundprinzip dauerhaft aktiv zu halten.

Die EU-Institutionen sind gefragt, tragfähige Lösungen zu diskutieren, und zwar zentral im Rahmen der Konferenz zur Zukunft Europas. Zudem sollte die Task Force Subsidiarität an ihre Vorschläge anknüpfen und ihre Arbeiten weiterführen. Wenn die EU-Institutionen diesen Weg nicht gehen, dann sollten zumindest entsprechende Impulse aus Politik und Wissenschaft formuliert werden, um Eingang in die Debatte zu finden. Nur relevante Subsidiarität in den Mitgliedstaaten begründet eine „aktive Subsidiarität“ in der EU.
